# Functional near-infrared spectroscopy study of intermittent theta burst stimulation on lower-limb motor dysfunction in patients with post-stroke hemiplegia

**DOI:** 10.3389/fnhum.2026.1849251

**Published:** 2026-06-22

**Authors:** Rui Jiang, Shuang Gong, Yu Zhao, Mingyu Wei, Hui Lin, Danfeng Zhang, Ronghong Shu

**Affiliations:** Department of Rehabilitation, Ningbo Rehabilitation Hospital, Ningbo, Zhejiang, China

**Keywords:** functional near-infrared spectroscopy, intermittent theta burst stimulation, lower limb, premotor cortex, stroke

## Abstract

**Objectives:**

Stroke is characterized by high morbidity and high disability rate, and is the main cause of long-term disability. To evaluate the efficacy of intermittent theta burst stimulation (iTBS) on patients with lower limb motor dysfunction after stroke through functional near-infrared spectroscopy (fNIRS) and the Fugl-Meyer Assessment of Lower Extremity Motor Function Scale, and analyze the activation of each channel of the cerebral cortex through fNIRS.

**Methods:**

Sixty-six patients were randomly assigned (1:1) to a control group (*n* = 33) or an iTBS treatment group (*n* = 33). The control group was given basic rehabilitation treatment and sham stimulation of iTBS, while the treatment group was given basic rehabilitation treatment and real stimulation of iTBS.

**Results:**

After treatment, when comparing the treatment group and the control group, there was a statistically significant difference in the scores of the Fugl-Meyer Lower Limb Motor Function Assessment Scale. After 4 weeks of treatment, when comparing the treatment group and the control group, there were statistically significant differences in task-related activation in channels over the ipsilesional premotor cortex (PMC) and primary motor cortex (M1), and the contralesional primary somatosensory cortex (S1) (*p* < 0.05). In the treatment group, task-related activation in channels over ipsilesional PMC and M1 and contralesional S1 was also significantly increased compared with baseline (*p* < 0.05).

**Conclusion:**

iTBS has a certain curative effect in the treatment of lower limb dysfunction after stroke. On fNIRS, increased activation was observed in channels over ipsilesional PMC and M1 and contralesional S1.

## 1 Preface

Stroke is characterized by high morbidity and high disability rate, and is the main cause of long-term disability (Jia et al., 2024). After rehabilitation treatment, more than 50% of patients will still have lower limb motor dysfunction (Shiwen et al., 2023). Lower limb dysfunction mainly involves weakened lower limb motor function. Long-term wheelchair life not only affects the psychological emotions of patients but also reduces the quality of life of patients (Shiheng et al., 2023). Insufficient muscle strength in the lower limbs of patients becomes the biggest inducement for falls in the future (Linhong et al., 2023), bringing greater mental stress and care costs to patients and family caregivers (Barral et al., 2021). How to carry out scientific treatment and rehabilitation for lower limb function after stroke, promote better recovery of lower limb function, and enable patients to better integrate into families and society has become a hot issue urgently to be solved in the rehabilitation medical field.

Among neuromodulation techniques for post-stroke motor recovery, intermittent theta burst stimulation (iTBS) is a patterned form of repetitive transcranial magnetic stimulation (rTMS) that has attracted increasing attention. iTBS is a new treatment mode of rTMS. It has the advantages of shorter treatment duration and similar or greater neuromodulatory efficacy compared with conventional high-frequency rTMS protocols, and is closer to the physiological rhythm of the central nervous system. It induces more lasting excitatory changes in the brain in a shorter time and effectively regulates neural plasticity (Song et al., 2020). Most clinical iTBS protocols deliver 600 pulses in 3–10 min at approximately 80% of the active motor threshold, using 50 Hz bursts repeated at 5 Hz. In this trial, we adopted a standard 600-pulse iTBS protocol consisting of 2-s trains of bursts followed by 8-s pauses, to match commonly used settings in post-stroke neuromodulation studies (Suppa et al., 2016). iTBS has been proven to induce long-term potentiation effects and can increase the excitability of the primary motor cortex, and can improve post-stroke motor dysfunction (Min et al., 2023). iTBS can affect behavior by promoting the ability of motor relearning through regional excitatory initiation. That is, if related synapses are reused in subsequent movements, iTBS may promote their strengthening (Butts et al., 2014; Platz et al., 2018). iTBS can change the cortical excitability of the stimulated site, as well as the excitability of its surrounding areas and even areas with distant connections (Li et al., 2020). iTBS can achieve similar therapeutic effects as rTMS and can reduce adverse reactions and save treatment time for patients. Based on the advantages of iTBS compared to rTMS, the iTBS treatment plan can be considered first.

To objectively monitor cerebral cortical changes induced by iTBS and rehabilitation, functional near-infrared spectroscopy (fNIRS) has been widely used in neurological research in recent years. fNIRS is a non-invasive optical technique that uses near-infrared light (700–1,100 nm) to measure changes in oxyhemoglobin (HbO₂) and deoxyhemoglobin (HbR) in the superficial cortex based on the principle of neurovascular coupling (Attwell et al., 2010; Bonvento and Bolanos, 2021; Butler et al., 2020; Guérin et al., 2021; Jia et al., 2023; Khan et al., 2022; Pinti et al., 2020). Compared with functional magnetic resonance imaging (fMRI), fNIRS is more portable, less sensitive to movement, and can be applied during overground or lower-limb tasks; compared with electroencephalography (EEG), it directly reflects hemodynamic changes related to cortical activation. These features make fNIRS particularly suitable for monitoring cortical reorganization in stroke patients with motor dysfunction during gait and lower-limb rehabilitation (Li et al., 2017; Li et al., 2019). Therefore, we selected fNIRS in this study to quantify task-related changes in motor and somatosensory cortex activation during lower-limb movements in stroke patients receiving iTBS.

iTBS can increase the excitability of the cerebral cortex, but there are few studies on the activation degree of each cortical channel for lower limb motor function in patients after stroke. Unlike previous iTBS studies focusing on upper limb recovery or using fMRI/EEG, this study specifically targets lower limb dysfunction using fNIRS—a modality uniquely suited for monitoring cortical activity during functional lower-limb tasks (Chen et al., 2025; Zhang et al., 2026; Zhou et al., 2025). Our channel-wise analysis provides spatially resolved insights into task-related activation patterns that may inform personalized rehabilitation. Therefore, in this study, we used fNIRS to conduct a comparative analysis on patients with lower limb motor disorders after stroke with and without iTBS treatment. This study aimed to provide a theoretical basis for improving rehabilitation strategies and objective evaluation methods for lower-limb motor dysfunction after stroke. Based on evidence that iTBS over the primary motor cortex enhances corticospinal excitability and improves motor performance, we hypothesized that adding iTBS over the ipsilesional primary motor cortex (M1) to conventional rehabilitation would (1) produce greater improvements in lower-limb motor function, as measured by the Fugl-Meyer Assessment of Lower Extremity (FMA-LE), and (2) be associated with increased task-related activation in ipsilesional premotor cortex (PMC)/M1 and contralesional primary somatosensory cortex (S1) as measured by fNIRS.

## 2 Materials and methods

### 2.1 Research subjects

**Figure 1:**
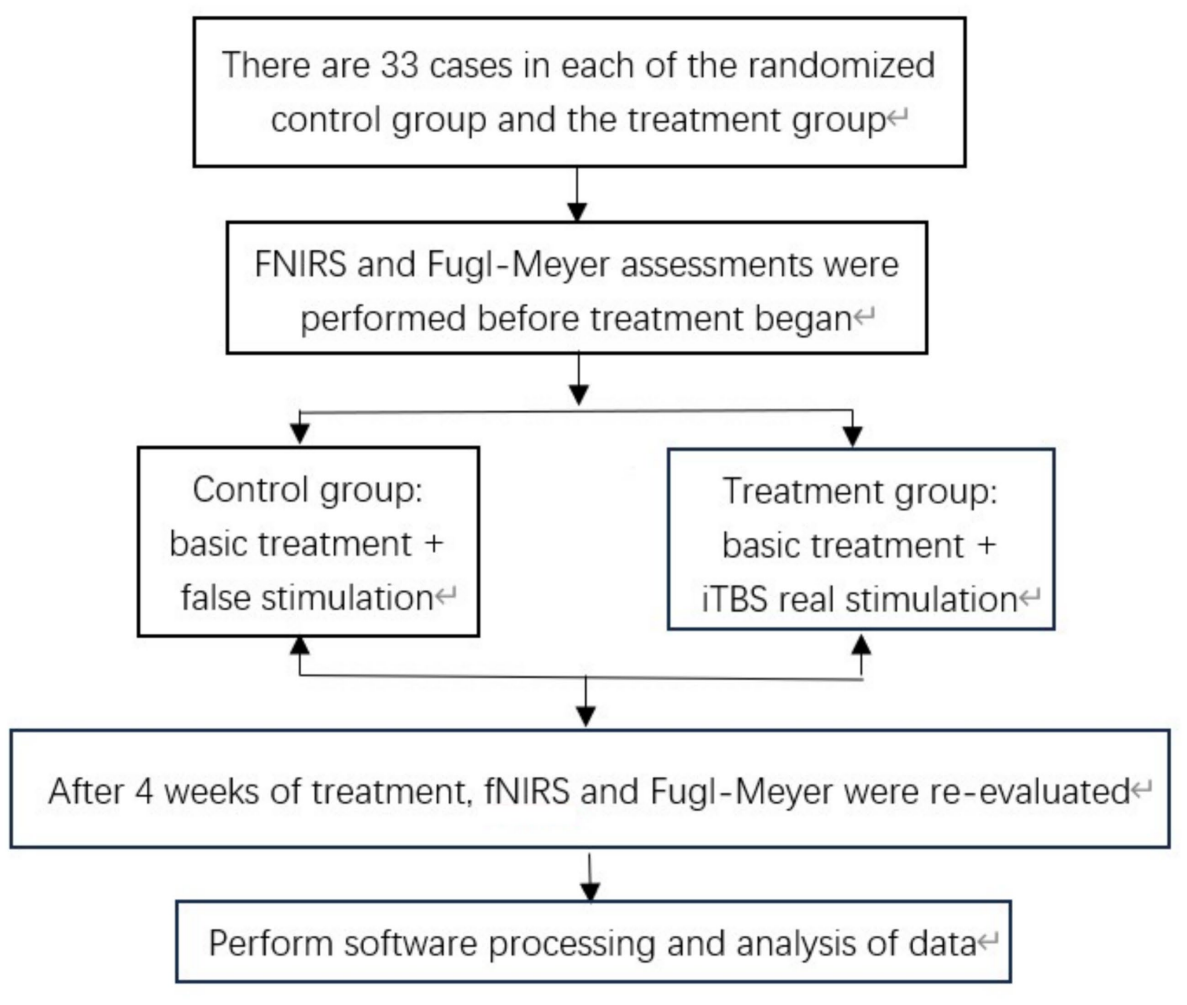
Research flow chart.

This study was reported to adhere to Consolidated Standards of Reporting Trials (CONSORT) guidelines (Supplementary File 1). A total of 66 stroke patients admitted to the rehabilitation department of Ningbo Rehabilitation Hospital who met the inclusion criteria were selected as the research objects. Using the random number table method, they were randomly divided into a treatment group and a control group, with 33 cases in each group (Figure 1). The random allocation sequence was generated by an investigator not involved in participant enrollment or outcome assessment using R software. Participants were enrolled by rehabilitation physicians who assigned each patient to the treatment or control group using sequentially numbered, opaque, sealed envelopes containing the group allocation. Outcome assessors were blinded to group assignment. Due to the nature of iTBS, participants could not be blinded to the presence of coil sensation, but sham stimulation (reversed coil) produced an identical sound and scalp sensation.

There was no statistically significant difference in general data such as gender composition, average age, and disease between the two groups (*p* > 0.05), and they were comparable (Table 1).

**Table 1:** Comparison of general information between the two groups.

Indicator		Control group	Treatment group	*p* value
Gender (example %)	Male	17 (51.5)	18 (54.5)	0.805
	female	16 (48.5)	15 (45.5)	
Stroke type (example %)	Cerebral infarction	19 (57.6)	18 (54.5)	0.804
	Cerebral hemorrhage	14 (42.4)	15 (45.5)	
Age (x ± s years old)		63.21 ± 12.07	61.12 ± 13.17	0.504
course of disease (x ± s day)		32.39 ± 16.96	33.15 ± 15.93	0.852

Inclusion criteria:

1. The diagnosis of stroke conforms to the diagnostic criteria of cerebrovascular disease formulated by the Chinese Medical Association in 1995. Computed tomography (CT) or magnetic resonance imaging (MRI) of the brain conforms to the clinical diagnosis of cerebral infarction or cerebral hemorrhage.
2. Left cerebral hemisphere stroke with right-sided hemiparesis.
3. First onset, stable condition, 2 weeks < course < 12 weeks, age 18 to 82 years old. We restricted the age to 18–82 years to ensure safer application of iTBS and reliable participation in task-based fNIRS assessment, because very elderly patients often have multiple comorbidities and difficulty completing repeated lower-limb tasks.
4. The condition is stable and can cooperate with rehabilitation training.
5. All patients themselves or their entrusted family members sign the informed consent form.

Exclusion criteria:

1. Other cases that do not meet the above inclusion criteria.
2. Have a history of epilepsy or are taking anti-epileptic drugs.
3. Wear a pacemaker, intracranial metal implants, and other metal implants in the body, or have a skull defect.
4. Severe higher brain dysfunction (for example, global aphasia, severe cognitive impairment, neglect, or apraxia) that prevented understanding instructions or completing the lower-limb task.

The relevant ethics of the participants involved in this study were reviewed and approved by the Ethics Committee of Ningbo Rehabilitation Hospital (Approval NO.: 2022–010), all methods were in accordance with the Declaration of Helsinki. Written informed consent was obtained from the individuals for the publication of any identifiable images or data contained in this article.

At the same time, all patients received guideline-based medical management for stroke, including antiplatelet or anticoagulant therapy, statins, antihypertensive medications, and other drugs as clinically indicated. As far as possible, no new medications known to directly influence cortical excitability (such as benzodiazepines or additional antiepileptic drugs) were initiated or discontinued during the intervention period.

No formal *a priori* sample size calculation was performed; the sample size was based on feasibility and the typical number of eligible patients within the recruitment period. Consequently, the trial may be underpowered for some exploratory fNIRS outcomes.

### 2.2 Methods

#### 2.2.1 Treatment method

Both groups of patients were given basic drugs and rehabilitation treatment for stroke.

##### 2.2.1.1 Treatment group

Based on basic treatment, iTBS therapy was added. The patient relaxed completely and sat in a sitting position. The center of the magnetic stimulation coil was aligned with the ipsilesional primary motor cortex (M1) hotspot for the paretic limb, corresponding approximately to C3 or C4 in the international 10–20 system. Although the lower-limb representation is closer to Cz, we targeted the conventional M1 hand-area hotspot to ensure reliable motor-threshold determination and consistent coil positioning, which has been shown to modulate broader motor networks in post-stroke patients. Treatment parameters: Three pulses of 50 Hz form a cluster. Each cluster is stimulated repeatedly at 5 Hz. Stimulation intensity was set to 80% of the resting motor threshold (RMT) of the affected lower limb. A standard figure-8-shaped coil (70 mm diameter) was used, with the coil close to the scalp, handle facing posterolaterally at a 30°–45° angle to the sagittal midline, producing a posterior–anterior induced current perpendicular to the cortical columns of the lower limb motor cortex. The intervention target was the M1 of the lower limb on the hemiplegic side. A two-step localization method was used: (1) anatomical positioning based on the 10/20 system (Cz point, then 1–2 cm lateral and posterior to the lesion side); (2) motion hotspot verification by identifying the site with the largest MEP amplitude and shortest latency in the tibialis anterior and rectus femoris muscles. When MEP could not be elicited, the anatomical point was used. Single iTBS session: 50 Hz, 3 pulses per cluster, 5 Hz cluster frequency, 2 s stimulation followed by 8 s interval, 20 cycles, total 600 pulses, duration 3 min 20 s. Treatment schedule: once daily, 5 days per week for 4 weeks (20 sessions total), with ≥24 h between sessions. Safety monitoring: contraindications screened (intracranial metal implants, epilepsy history, severe headache history); during treatment, patients were monitored for scalp tingling, headache, dizziness, limb numbness, convulsions, and seizure signs. Minor adverse reactions (localized scalp discomfort, brief headache) were self-limiting and recorded.

##### 2.2.1.2 Control group

Based on basic treatment, sham stimulation with iTBS is given. The sham stimulation time and frequency are the same as those in the treatment group. The coil is reversed and placed on the surface of the patient’s head to form the same stimulation sound, which is an ineffective stimulation.

Both groups of patients were treated for 4 weeks, with 5 days of treatment per week. At the same time, basic rehabilitation training was given. Before treatment and after 4 weeks of treatment, the FMA-LE Motor Function Scale and fNIRS assessment were performed, respectively.

#### 2.2.2 Evaluation of lower limb function

The FMA-LE Motor Function Scale was used to assess the motor function of the affected lower limb before and after treatment in the two groups of patients (Beckerman et al., 1996). This scale requires patients to perform a series of actions to check the limb reflex state, flexion-extension synergistic movement, and selective separation movement, including seven major items such as reflex, flexor, and extensor synergistic movement, activities accompanied by synergistic movement, separation movement, hyperreflexia, coordination ability, and speed, and 19 minor items. The grading is divided into three levels (0–2 points), and the total score is 38 points. The higher the score, the better the motor function of the patient.

#### 2.2.3 Data acquisition and processing of fNIRS

**Figure 2:**
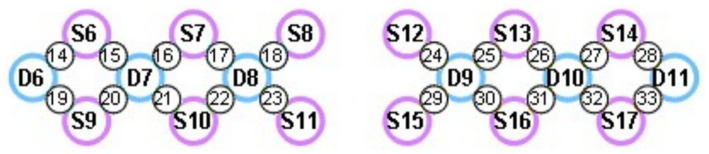
fNIRS brain monitoring channels.

In this study, a multi-channel, portable fNIRS device (NirSmart-6000A, Hui chuang Medical Equipment Co. Ltd., Danyang, China) was used to record changes in HbO₂. The device is located according to the 10/20 international standard lead system. This study consists of 18 light source probes and 18 detection probes to form 20 effective channels (Figure 2). The detection areas include the bilateral primary sensory cortex (S1), primary motor cortex (M1), supplementary motor area (SMA), and premotor area (PMC). The distance between the receiving probe and the transmitting probe of the head cap is 3 cm. The probe materials are all avalanche photodiodes. Near-infrared light (695, 830 nm) signals of two different wavelengths are recorded in continuous wave form, and the sampling frequency is 11 Hz.

Channels were assigned to three regions of interest (ROIs)—primary motor cortex (M1), premotor cortex (PMC), and primary somatosensory cortex (S1)—based on their position relative to 10–20 landmarks (Cz, C3/C4, CP3/CP4, FC3/FC4). The montage and ROI allocation are illustrated in Figure 2.

##### 2.2.3.1 Design of fNIRS test task

Preparation stage: In a quiet room with only the tester and the subject, instruct the subject to maintain good sleep and a normal mental state before the test, and avoid interference from irritating foods and drugs. Before the test, the subject should be trained for the test task and start the test after sufficient rest.

**Figure 3:**
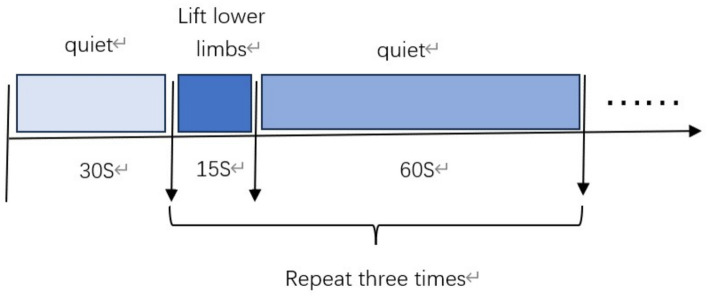
Schematic diagram of the lower limb lift task.

Testing stage: The subject lies in the supine position and keeps the rest of the body still except for the movement of the affected lower limb (complete the test movements as much as possible without compensation in other parts). Head movement and speaking are prohibited, and distractions are excluded. The tester should give simple and accurate language prompts to the subject at the start and stop of the task. Test movement: After the start, the affected lower limb of the subject begins to perform hip flexion, knee flexion, and dorsiflexion of the foot. During each 15-s active block, patients repeatedly performed a continuous sequence of these right lower-limb movements—hip flexion, knee flexion, and ankle dorsiflexion—in a cyclical pattern at a comfortable pace, without compensatory trunk or upper-limb movements. The test uses block stimulation. During the test, the activity sequence of the affected lower limb of the subject is as follows: rest for 30 s → activity for 15 s → rest for 60 s → activity for 15 s → rest for 60 s → activity for 15 s → rest for 60 s. This cycle is repeated three times in total (Figure 3). The 15-s activity block, therefore, reflects the integrated hemodynamic response to a functional multi-joint lower-limb task rather than isolated single-joint movements.

##### 2.2.3.2 fNIRS data processing

The NirSpark software (Hui chuang Medical Equipment Co. Ltd., Danyang, China) is used to process and analyze the near-infrared brain functional imaging data.

Step 1: Pretreatment. Convert the original light intensity data into optical density data. Raw intensity data were band-pass filtered at 0.01–0.1 Hz to remove slow drifts and cardiac/respiratory components. Set the signal standard deviation threshold to 6 and the peak threshold to 0.5. Use spline interpolation to correct motion artifacts. According to the modified Beer–Lambert law, using wavelength-specific differential pathlength factors (for example, 6.0 at 695 nm and 5.2 at 830 nm), convert the filtered optical density data into the concentrations of HbO₂ and HbR. Since the noise ratio of HbO₂ is better than that of HbR (Strangman et al., 2002) and HbO₂ is more sensitive to regional cerebral blood flow response than HbR (Bai et al., 2020), we selected the HbO₂ value for data analysis. At the same time, deoxyhemoglobin (HbR) time courses were examined to verify the expected inverse relationship relative to HbO₂ during task-related activation, ensuring physiological consistency of the hemodynamic response. All preprocessing procedures were conducted using the standardized manufacturer pipeline in NirSpark, which is conceptually comparable to established fNIRS processing workflows such as Homer2/Homer3. No additional manual adjustments or signal manipulations were performed beyond the predefined preprocessing steps described above.

Step 2: After pretreatment, perform block average analysis on the task. Use the task block of the first 15 s. Obtain the mean value of HbO₂ concentration of different channels by editing the eigenvalue. To compare channel-wise HbO₂ changes between and within groups, we used the Mann–Whitney U test and the Wilcoxon signed-rank test. To control for multiple comparisons across channels, *p*-values were adjusted using the Benjamini–Hochberg false discovery rate (FDR) procedure, with q < 0.05 considered statistically significant. For non-parametric tests, we report the test statistic (Z or W), effect size r (r = Z/√N), and 95% confidence intervals where appropriate. For both test methods, the significance threshold is set as *p* < 0.05.

### 2.3 Statistical analysis

In addition to the analysis by NirSpark software, other data also need to be statistically analyzed using SPSS 26.0 software. The chi-square test is used for gender and stroke type. Normality of continuous variables was assessed using the Shapiro–Wilk test. Age and course of disease are in normal distribution, and an independent sample t-test is used. The scores of the FMA-LE Motor Function Scale and fNIRS data are not in normal distribution, and the Mann–Whitney test and the Wilcoxon signed-rank sum test are used. For each channel and for FMA-LE, we conducted within-group analyses (pre vs. post) and between-group analyses (treatment vs. control) at each time point. Given the modest sample size and the exploratory nature of the fNIRS analyses, we did not fit higher-order mixed-effects models but instead treated the channel-wise comparisons as exploratory analyses corrected for multiple testing using the FDR procedure described above. *p* < 0.05 indicates statistically significant differences.

To explore the relationship between cortical hemodynamic changes and clinical motor improvement, correlation coefficients were calculated between the change scores (post-treatment minus pre-treatment) of the FMA-LE and the change in HbO₂ concentration in each significant fNIRS channel in the treatment group. Correlation coefficients and two-tailed *p* values were reported. Given the exploratory nature of this analysis, no additional adjustment for multiple comparisons was applied, and results should be interpreted cautiously. We also performed exploratory subgroup analyses based on sex (male vs. female) and stroke subtype (cerebral infarction vs. intracerebral hemorrhage). For each subgroup, we compared the between-group difference in FMA-LE scores and fNIRS channels using the Mann–Whitney U test.

## 3 Result

### 3.1 Baseline characteristics

The baseline demographic and clinical characteristics of the two groups are summarized in Table 1. There were no statistically significant differences between the treatment group and the control group in terms of age, sex distribution, stroke type (cerebral infarction vs. cerebral hemorrhage), or time since stroke onset (all *p* > 0.05), indicating good baseline comparability. Both groups received the same institutional conventional rehabilitation program in addition to their assigned real or sham iTBS, as detailed in the Methods.

### 3.2 Changes in lower-limb motor function

At baseline, FMA-LE scores did not differ significantly between the treatment and control groups [median (Q1–Q3) 17 (15–23) vs. 15 (13–18), *p* = 0.168; Table 2]. Within each group, FMA-LE scores improved significantly after 4 weeks of intervention (both *p* < 0.001; Table 3). After treatment, the treatment group showed markedly higher FMA-LE scores than the control group [median (Q1–Q3) 26 (22–29) vs. 19 (17–23), *p* < 0.001; Table 2], indicating greater gains in lower-limb motor function when iTBS was added to conventional rehabilitation.

**Table 3:** Comparison of Fugl-Meyer Assessment of Lower Extremity Motor Function Scale scores within the control group and the treatment group.

Group	Time	Median (Q1-Q3)	*p* value	Effect size
Control group	Before treatment	15 (13–18)	<0.001	−0.433
	After treatment	19 (17–23)		
Treatment group	Before treatment	17 (15–23)	<0.001	−0.573
	After treatment	26 (22–29)		

**Table 2:** Comparison of Fugl-Meyer Assessment of Lower Extremity Motor Function Scale scores between the control group and the treatment group.

Time	Treatment group	Control group	*p* value	Effect size
	Median (Q1-Q3)	Median (Q1-Q3)		
Before treatment	17 (15–23)	15 (13–18)	0.168	−0.171
After treatment	26 (22–29)	19 (17–23)	<0.001	−0.419

### 3.3 Task-related cortical activation measured by fNIRS

#### 3.3.1 Within-group comparisons

Before treatment, there were no statistically significant task-related differences in HbO₂ activation across any channels within either the control or treatment group. After 4 weeks of intervention, the control group still showed no significant changes in cortical activation in any channel [all *q* ≥ 0.05 after false discovery rate (FDR) correction; Table 4; Figure 4A]. In contrast, the treatment group demonstrated significantly increased HbO₂ responses in channels overlying the ipsilesional premotor cortex (PMC; S7–D8, S8–D8), ipsilesional primary motor cortex (M1; S10–D8, S11–D8), and contralesional primary somatosensory cortex (S1; S17–D10) (all *q* < 0.05; Table 4; Figure 4B).

**Table 4:** The P values of the within-group comparisons before and after treatment in the control group and the treatment group.

Channel	S-D	Brain region	Control group				Treatment group			
			Before treatment/after treatment median (Q1-Q3)	*p* value	Effect size	*q* value	Before treatment/after treatment median (Q1-Q3)	*p* value	Effect size	*q* value
14	S6-D6	S1	0.03 (0.01–0.07)	0.195	−0.160	0.834	0.04 (0.02–0.07)	0.063	−0.230	0.207
			0.03 (0.02–0.09)				0.05 (0.04–0.07)			
15	S6-D7	PMC	0.02 (0.01–0.07)	0.171	−0.170	0.834	0.04 (0.02–0.05)	0.744	−0.041	0.778
			0.03 (0.02–0.06)				0.03 (0.01–0.08)			
16	S7-D7	PMC	0.03 (0.02–0.06)	0.665	−0.054	0.848	0.06 (0.03–0.07)	0.744	−0.041	0.778
							0.04(0.02–0.12)			
			0.02 (0.02–0.06)							
17	S7-D8	PMC	0.04 (0.02–0.05)	0.770	−0.037	0.848	0.02 (0.01–0.05)	0.028#	−0.271	0.131
			0.03 (0.01–0.07)				0.05 (0.04–0.08)			
18	S8-D8	PMC	0.04 (0.03–0.09)	0.358	−0.115	0.834	0.06 (0.03–0.11)	0.002#	−0.369	0.027
			0.05 (0.04–0.09)				0.1(0.04–0.17)			
19	S9-D6	S1	0.03 (0.02–0.06)	0.483	−0.088	0.834	0.02 (0.02–0.05)	0.665	−0.054	0.774
			0.03 (0.01–0.06)				0.02 (0.02–0.1)			
20	S9-D7	S1	0.04 (0.02–0.06)	0.619	−0.062	0.848	0.03 (0.02–0.06)	0.225	−0.151	0.368
			0.04 (0.01–0.07)				0.04 (0.02–0.06)			
21	S10-D7	S1	0.02 (0–0.05)	0.473	−0.089	0.834	0.04 (0.02–0.04)	0.192	−0.162	0.368
							0.04 (0.01–0.07)			
			0.04 (0.01–0.07)							
22	S10-D8	M1	0.04 (0.02–0.08)	0.215	−0.154	0.834	0.04 (0.02–0.05)	0.033#	−0.263	0.131
							0.05 (0.03–0.1)			
			0.03 (0.02–0.1)							
23	S11-D8	M1	0.07 (0–0.09)	0.740	−0.042	0.848	0.03 (0.02–0.03)	0.011#	−0.310	0.078
			0.03 (0.02–0.05)				0.05 (0.03–0.08)			
24	S12-D9	PMC	0.02 (0.02–0.03)	0.532	−0.078	0.834	0.02 (0.01–0.08)	0.397	−0.105	0.525
							0.03 (0.02–0.06)			
			0.03 (0.02–0.05)							
25	S13-D9	PMC	0.02 (0.02–0.08)	0.693	−0.050	0.848	0.05 (0.02–0.07)	0.566	−0.072	0.7
			0.03 (0.02–0.04)				0.04 (0.03–0.08)			
26	S13-D10	PMC	0.04 (0.01–0.06)	0.179	−0.167	0.834	0.04 (0.01–0.06)	0.949	−0.009	0.944
			0.03 (0.01–0.04)				0.03 (0.01–0.08)			
27	S14-D10	PMC	0.06 (0.01–0.1)	0.848	−0.025	0.886	0.04 (0.02–0.05)	0.148	−0.179	0.324
			0.05 (0.01–0.1)				0.05 (0.02–0.09)			
28	S14-D11	S1	0.04 (0.02–0.09)	0.97	−0.006	0.964	0.05 (0.02–0.06)	0.141	−0.182	0.324
			0.05 (0.03–0.06)				0.06 (0.04–0.09)			
29	S15-D9	M1	0.05 (0.04–0.06)	0.383	−0.108	0.834	0.04 (0.02–0.13)	0.077	−0.219	0.216
			0.06 (0.01–0.1)				0.08 (0.04–0.14)			
30	S16-D9	M1	0.05 (0.04–0.06)	0.46	−0.092	0.834	0.05 (0.04–0.08)	0.365	−0.113	0.513
			0.05 (0.02–0.07)				0.06 (0.05–0.1)			
31	S16-D10	S1	0.02 (0.02–0.08)	0.483	−0.088	0.834	0.04 (0.02–0.06)	0.243	−0.144	0.371
			0.05 (0.01–0.08)				0.05 (0.03–0.08)			
32	S17-D10	S1	0.03 (0.02–0.05)	0.422	−0.100	0.834	0.02 (0.01–0.07)	<0.001#	−0.435	0.008
			0.03 (0.03–0.07)				0.05 (0.04–0.09)			
33	S17-D11	S1	0.04 (0.01–0.06)	0.549	−0.075	0.834	0.03 (0.01–0.08)	0.220	−0.152	0.368
			0.07 (0.01–0.1)				0.05 (0.03–0.1)			

#*p* < 0.05.

**Figure 4:**
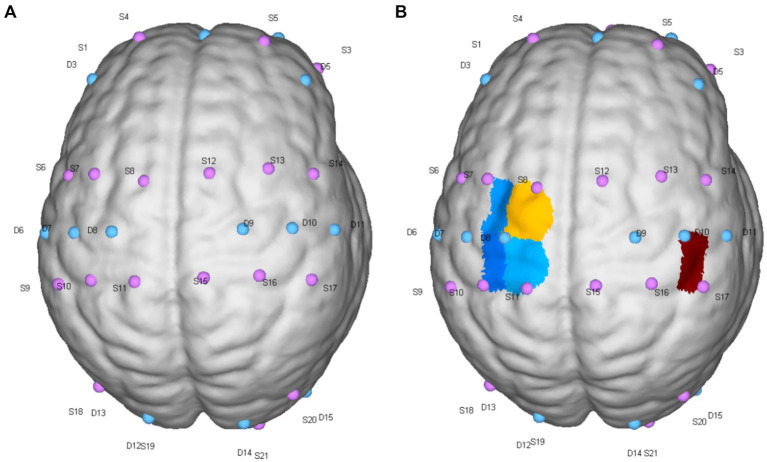
Display diagram of the activated channels for the within-group comparison before and after treatment in the two groups. **(A)** Before and after treatment in the control group; **(B)** Before and after the treatment in the treatment group.

#### 3.3.2 Between-group comparisons

There were no significant between-group differences in HbO₂ activation in any channel at baseline (all *q* ≥ 0.05; Table 5; Figure 5A). After 4 weeks, however, the treatment group showed significantly greater task-related activation than the control group in channel S8–D8 over PMC, channels S10–D8 and S11–D8 over M1, and channel S17–D10 over S1 (all *q* < 0.05; Table 5; Figure 5B).

**Table 5:** The *p* values of the between-group comparisons before and after treatment in the control group and the treatment group.

Channel	S-D	Brain region	Before treatment				After treatment			
			Treatment/control median (Q1-Q3)	*p* value	Effect size	*q* value	Treatment/control median (Q1-Q3)	*p* value	Effect size	*q* value
14	S6-D6	S1	0.04 (0.02–0.07)	0.521	−0.080	0.795	0.05 (0.04–0.07)	0.197	−0.160	0.483
							0.03 (0.02–0.09)			
			0.03 (0.01–0.07)							
15	S6-D7	PMC	0.04 (0.02–0.05)	0.429	−0.099	0.795	0.03 (0.01–0.08)	0.889	−0.018	0.883
			0.02 (0.01–0.07)				0.03 (0.02–0.06)			
16	S7-D7	PMC	0.06 (0.03–0.07)	0.104	−0.201	0.733	0.04 (0.02–0.12)	0.358	−0.115	0.622
			0.03 (0.02–0.06)				0.02 (0.02–0.06)			
17	S7-D8	PMC	0.02 (0.01–0.05)	0.407	−0.103	0.795	0.05 (0.04–0.08)	0.175	−0.168	0.483
			0.04 (0.02–0.05)				0.03 (0.01–0.07)			
18	S8-D8	PMC	0.06 (0.03–0.11)	0.483	−0.088	0.795	0.1 (0.04–0.17)	<0.001#	−0.446	0.006
			0.04 (0.03–0.09)				0.05 (0.04–0.09)			
19	S9-D6	S1	0.02 (0.02–0.05)	0.33	−0.121	0.795	0.02 (0.02–0.1)	0.656	−0.056	0.721
			0.03 (0.02–0.06)				0.03 (0.01–0.06)			
20	S9-D7	S1	0.03 (0.02–0.06)	0.112	−0.197	0.733	0.04 (0.02–0.06)	0.566	−0.072	0.700
			0.04 (0.02–0.06)				0.04 (0.01–0.07)			
21	S10-D7	S1	0.04 (0.02–0.04)	0.345	−0.118	0.795	0.04 (0.01–0.07)	0.608	−0.064	0.709
			0.02 (0–0.05)				0.04 (0.01–0.07)			
22	S10-D8	M1	0.04 (0.02–0.05)	0.283	−0.133	0.795	0.05 (0.03–0.1)	0.049#	−0.242	0.245
			0.04 (0.02–0.08)				0.03 (0.02–0.1)			
23	S11-D8	M1	0.03 (0.02–0.03)	0.610	−0.064	0.841	0.05 (0.03–0.08)	0.007#	−0.331	0.048
			0.07 (0–0.09)				0.03 (0.02–0.05)			
24	S12-D9	PMC	0.02 (0.01–0.08)	0.729	−0.043	0.845	0.03 (0.02–0.06)	0.779	−0.036	0.814
			0.02 (0.02–0.03)				0.03 (0.02–0.05)			
25	S13-D9	PMC	0.05 (0.02–0.07)	0.809	−0.031	0.845	0.04 (0.03–0.08)	0.319	−0.124	0.622
							0.03 (0.02–0.04)			
			0.02 (0.02–0.08)							
26	S13-D10	PMC	0.04 (0.01–0.06)	0.939	−0.010	0.934	0.03 (0.01–0.08)	0.379	−0.110	0.622
							0.03 (0.01–0.04)			
			0.04 (0.01–0.06)							
27	S14-D10	PMC	0.04 (0.02–0.05)	0.278	−0.135	0.795	0.05 (0.02–0.09)	0.25	−0.143	0.547
			0.06 (0.01–0.1)				0.05 (0.01–0.1)			
28	S14-D11	S1	0.05 (0.02–0.06)	0.637	−0.059	0.841	0.06 (0.04–0.09)	0.475	−0.089	0.625
			0.04 (0.02–0.09)				0.05 (0.03–0.06)			
29	S15-D9	M1	0.04 (0.02–0.13)	0.449	−0.094	0.795	0.08 (0.04–0.14)	0.101	−0.203	0.397
			0.05 (0.04–0.06)				0.06 (0.01–0.1)			
30	S16-D9	M1	0.05 (0.04–0.08)	0.809	−0.031	0.845	0.06 (0.05–0.1)	0.121	−0.192	0.397
			0.05 (0.04–0.06)				0.05 (0.02–0.07)			
31	S16-D10	S1	0.04 (0.02–0.06)	0.063	−0.230	0.733	0.05 (0.03–0.08)	0.422	−0.100	0.625
			0.02 (0.02–0.08)				0.05 (0.01–0.08)			
32	S17-D10	S1	0.02 (0.01–0.07)	0.261	−0.140	0.795	0.05 (0.04–0.09)	0.005#	−0.342	0.048
			0.03 (0.02–0.05)				0.03 (0.03–0.07)			
33	S17-D11	S1	0.03 (0.01–0.08)	0.789	−0.034	0.845	0.05 (0.03–0.1)	0.444	−0.095	0.625
							0.07 (0.01–0.1)			
			0.04 (0.01–0.06)							

#*p* < 0.05.

**Figure 5:**
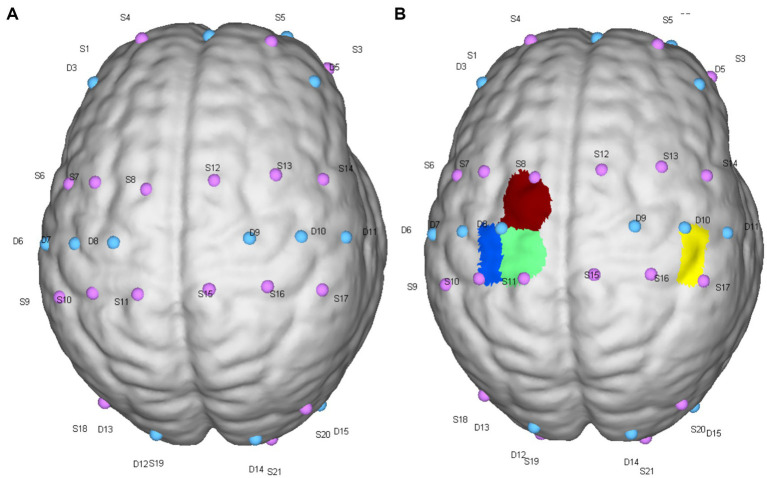
Display a diagram of the activated channels for the between-group comparison before and after treatment in the two groups. **(A)** Control group and treatment group before treatment; **(B)** Control group and treatment group after treatment.

#### 3.3.3 Summary of fNIRS findings

Overall, the fNIRS results indicate that iTBS combined with conventional rehabilitation was associated with enhanced task-related activation of the ipsilesional PMC and M1 and the contralesional S1 during lower-limb movements, whereas conventional rehabilitation alone did not produce comparable changes. Because the channel-wise analyses are exploratory and based on a modest sample size, even with FDR correction, these neuroimaging findings should be interpreted cautiously and viewed as hypothesis-generating.

#### 3.3.4 Exploratory analysis

In the treatment group, we examined the relationship between changes in HbO₂ (post-treatment minus pre-treatment) and changes in FMA-LE scores. Moderate positive correlations were observed for S6-D7 (*r* = 0.424, *p* = 0.014), S7-D7 (*r* = 0.361, *p* = 0.039), S9-D7 (*r* = −0.375, *p* = 0.031), S10-D7 (*r* = 0.375, *p* = 0.032), S16-D9 (*r* = −0.483, *p* = 0.004) (Supplementary Table 1). These post-hoc exploratory findings suggest an association between increased sensorimotor activation and motor improvement, but causality cannot be inferred. In all subgroup analyses, the direction of effect favored the iTBS group over the control group (Supplementary Tables 2–5). However, due to the modest sample size, we could not reliably assess effect modification by lesion location or baseline severity, and these findings should be considered hypothesis-generating.

## 4 Discussion

Lower limb motor disorders after stroke can reduce the quality of life, increase the risk of falls, and hinder the return to society (Bai et al., 2020; Beckerman et al., 1996; Grau-Pellicer et al., 2019; Li et al., 2017; Li et al., 2019; Minet et al., 2015; Strangman et al., 2002). In this study, patients with lower limb motor impairment after stroke were treated with iTBS (intermittent theta burst stimulation) in addition to conventional rehabilitation training. The results showed that, compared with the control group, the patients treated with iTBS had higher scores in the FMA-LE. In terms of fNIRS (functional near-infrared spectroscopy), the treatment group showed greater task-related activation in channels over the ipsilesional PMC and M1 and the contralesional S1 after 4 weeks of treatment, whereas the control group did not exhibit comparable changes. Although effect sizes for channel-wise HbO₂ changes were generally in the small-to-moderate range, the overall pattern of increased activation in ipsilesional PMC/M1 and contralesional S1 is consistent with enhanced recruitment of sensorimotor networks during lower-limb tasks. Taken together, these findings suggest that adding iTBS to conventional rehabilitation may facilitate the recruitment of sensorimotor regions during lower-limb movements and is associated with improved motor outcomes, although the present trial cannot establish definitive causality.

fNIRS is a non-invasive neuroimaging method that can detect changes in cerebral hemodynamics during task execution. Due to its safety, portability, and low cost, it is widely used in neuroscience research and clinical practice (Zhao et al., 2024). fNIRS has strong anti-motion artifact ability in a moving state and can be worn on the body, and is applied in the assessment of lower limb motor function (Youxin et al., 2023). fNIRS can monitor the activation of different brain regions in real time when patients perform specific tasks by detecting changes in cerebral hemodynamics. fNIRS can monitor cortical responses, provide real-time feedback, and establish evaluations of neural stimulation (Huo et al., 2024). Li et al. (2023) applied transcranial direct current stimulation combined with transcutaneous electrical nerve stimulation to treat shoulder pain symptoms in patients after stroke. Through a comparative study by fNIRS, it was found that there was a significant difference in the average HbO₂ concentration in PMC. Shiheng et al. (2023) found through fNIRS that the recovery of lower limb motor function may be related to the enhanced activation of the affected side PMC. Ma et al. (2022) found through fNIRS that the prefrontal cortex (PFC) and motor cortex (MC) regions were activated during the functional neural stimulation task period. Therefore, many studies have shown that fNIRS can be used to evaluate the activation of different brain regions when patients perform specific tasks.

In fNIRS, PMC is the premotor area, M1 is the primary motor cortex area, and S1 is the primary sensory cortex area. In this study, the activation degree of the M1 and PMC channels on the affected side is stronger. This pattern is consistent with iTBS being applied over the ipsilesional M1 and modulating the excitability of surrounding motor-related cortical regions. iTBS can correct the pathological inhibitory pattern between the cerebral hemispheres (Yan et al., 2018). iTBS is an emerging non-invasive brain stimulation technology and has shown certain potential in treating lower limb movement disorders after stroke. Lower limb movement disorders after stroke can seriously affect the daily life and mobility of patients. By modulating cortical excitability, iTBS may help activate previously inhibited neural pathways and support motor relearning, thereby contributing to better control of lower-limb movements. After a stroke, the imbalance of the excitatory-inhibitory state of the brain causes abnormal motor function. Modulation of cortical excitability by iTBS may partially rebalance interhemispheric interactions and support motor recovery (Tian et al., 2022). iTBS can induce long-term potentiation and increase neural plasticity (Jialin et al., 2021; Zhigang et al., 2022). Stimulating the cerebral cortex can induce changes in neural plasticity, including the enhancement of synaptic connections, the formation of new neural connections, and the regeneration of nerve cells. This change in neural plasticity helps restore the damaged motor neural network and improve the motor function of the lower limbs. Shurui and Li (2022) found that suspension exercise training combined with cerebellar iTBS can significantly improve the lower limb motor function of stroke patients; Chen et al. (2019) found that iTBS treatment can improve the lower limb spasticity of patients; Sasaki et al. (2017) intervened with rTMS of different frequencies on stroke patients through a conical coil. The results showed that high-frequency rTMS treatment has a more positive effect on the recovery of lower limb motor function after stroke. Chen et al. (2023) found that iTBS significantly improved the motor impairment of stroke patients and reduced muscle tone, thereby improving the ability to perform activities of daily living.

M1 is located in the precentral gyrus and is directly related to limb movement. It is mainly responsible for the movement execution of the contralateral limb and generates motor command pulses to control the execution of movement through spinal cord conduction (Rivara et al., 2003). When the human body performs active movement, M1 will be activated, manifested as changes in local cerebral blood flow and blood oxygen level, which can be detected by fNIRS. The application of iTBS to M1 has been proven to regulate the excitability and connectivity of the motor system (Hensel et al., 2019). PMC is located on the lateral surface of the frontal lobe of the brain and is mainly responsible for motor planning and performing complex motor behaviors (Kantak et al., 2012). PMC plays an irreplaceable role in all key links of movement. Monitoring PMC with fNIRS can help us deeply understand the activation state of this area in different motor tasks. Lin et al. (2013) monitored the activation of the cerebral cortex during active and assisted riding in stroke patients through fNIRS, and the results showed that enhanced speed feedback was associated with activation of the cerebral cortical PMC area. Miyai et al. (2003) studied the cortical activation of stroke patients monitored by fNIRS in the walking task state and found that the recovery of lower limb motor function in stroke patients is greatly related to the enhanced activation of the affected side PMC. M1 and PMC participate in the process of initiating motor function by connecting with the brainstem and basal ganglia. The enhanced activation of these areas is related to the improvement of lower limb function. S1 is mainly responsible for receiving and processing sensory information from different parts of the body. When the body is stimulated externally, S1 will be activated. During movement, while M1 issues motor commands, it also receives sensory feedback from S1 to adjust the precision and strength of movement. Through fNIRS, the blood oxygen changes of M1 and S1 can be monitored simultaneously, and the functional connection between them can be studied, which helps to deeply understand the synergistic mechanism of the brain in motor control and sensory processing. The strong activation degree of the S17-D10 channel on the contralesional S1 is considered to be related to reasons such as brain plasticity and interhemispheric sensorimotor network connectivity. Min et al. (2023) found that the functional connection strength between the affected side M1 and the contralateral side S1 in the iTBS stimulation group of stroke patients with motor disorders is stronger than that in the placebo stimulation group. Some studies have found that the brain regions involved in motor function recovery after stroke are prominent in the activation of the sensorimotor area (Hannanu et al., 2017), which is similar to the high activation degree of the contralateral side S1 area in this study. For stroke patients, iTBS can regulate the excitatory imbalance between the damaged cerebral hemisphere and the contralateral cerebral hemisphere and promote the recovery of neurological function. Ding et al. (2021) also showed that iTBS can regulate the brain network function of stroke survivors and can improve the acute increase in interhemispheric functional connectivity and global efficiency. In line with these observations, our fNIRS results suggest that iTBS may facilitate recruitment of a broader sensorimotor network during lower-limb tasks, rather than acting only on a single cortical site.

The strong activation of the contralesional S1 (channel S17-D10) may reflect several non-mutually exclusive mechanisms: (1) adaptive compensatory recruitment to provide sensory feedback during lower-limb movements when ipsilesional S1 is damaged; (2) enhanced sensory-motor integration supporting motor relearning, as S1 provides critical afferent input to M1; (3) possible maladaptive reorganization if hyperactivation persists chronically; or (4) interhemispheric rebalancing following iTBS of the ipsilesional M1, modulating not only motor but also sensory cortical excitability. Post-stroke sensory network plasticity is increasingly recognized as important for motor recovery (Lu et al., 2025; Yao et al., 2025).

However, the interpretation of increased task-related HbO₂ must remain cautious. Increased task-related HbO₂ activation after stroke may reflect compensatory recruitment of sensorimotor regions rather than direct normalization of motor networks. Therefore, greater cortical activation should not necessarily be interpreted as uniformly beneficial recovery. In stroke, task-related hyperactivation in sensorimotor regions is often more pronounced in the subacute phase and may normalize as patients recover. Our findings of greater HbO₂ increases in the iTBS group are therefore more likely to reflect compensatory recruitment of sensorimotor areas during motor relearning than a simple linear “more activation is always better” relationship. Longitudinal studies with multiple follow-up time points are needed to determine whether these activation patterns eventually normalize as lower-limb function stabilizes.

While task-related HbO₂ changes in sensorimotor regions are promising as potential biomarkers of post-stroke plasticity, we did not validate these fNIRS measures against established neurophysiological indices such as TMS-derived corticospinal excitability (for example, motor-evoked potential amplitudes, short-interval intracortical inhibition, or intracortical facilitation) or EEG rhythms. Future studies integrating iTBS, fNIRS, and TMS/EEG, together with clinical scales, are needed to build a more robust multimodal framework for monitoring sensorimotor reorganization in stroke rehabilitation.

Similar neuromodulation strategies have also been applied to upper-limb rehabilitation after stroke. Previous studies demonstrated that iTBS combined with rehabilitation training can improve upper-limb motor recovery and cortical reorganization, suggesting that the therapeutic mechanisms observed in lower-limb rehabilitation may also extend to upper-limb motor networks (Dai et al., 2024; Liu et al., 2025; Wang et al., 2025).

From a translational perspective, integrating iTBS with fNIRS is feasible in clinical rehabilitation settings. The portable fNIRS device was well-tolerated, and the iTBS protocol required only 3 min 20 s per session, minimizing patient burden. The observed improvement in FMA-LE scores (median increase of 9 points in the treatment group vs. 4 points in controls) suggests that the statistical improvements are clinically meaningful. However, cost–benefit analysis and scalability to community rehabilitation settings require further study.

This study also has several limitations. First, this was a single-centre trial with a modest sample size, so the study may be underpowered for some exploratory fNIRS outcomes, particularly after correction for multiple comparisons. Second, although we applied FDR correction, the channel-wise analyses remain exploratory and should be confirmed in larger samples using model-based approaches and independent replication. Third, our block design involved multi-joint movements (hip, knee and ankle) within a 15-s window, which does not allow us to disentangle the contribution of individual joints to the observed cortical activation; single-joint tasks in future work may help clarify joint-specific hemodynamic responses. Fourth, the fNIRS montage sampled only superficial cortical regions, and regions of interest were assigned based on 10–20 landmarks rather than individual MRI, so deep and subcortical structures and precise anatomical borders could not be assessed. Fifth, we excluded patients with severe higher brain dysfunction that prevented task performance, and we attempted to keep medications that influence cortical excitability stable; nevertheless, residual variability in cognition and concomitant drugs may still have influenced both behavioural and fNIRS outcomes. Sixth, we did not perform detailed analyses of functional connectivity between cortical channels or construct whole-brain networks from the fNIRS data, and therefore cannot describe how iTBS influenced large-scale brain networks. Finally, although exploratory correlations between FMA-LE changes and channel-wise HbO₂ were performed, these analyses were limited and should be interpreted cautiously.

## 5 Conclusion

In this randomized controlled trial, adding iTBS over the ipsilesional M1 to conventional rehabilitation was associated with greater improvements in lower-limb motor function and increased task-related activation of ipsilesional PMC/M1 and contralesional S1 compared with conventional rehabilitation alone. fNIRS provided a practical and economical method for monitoring these cortical hemodynamic changes during lower-limb tasks. Given the modest sample size and the exploratory nature of the fNIRS analyses, these findings should be confirmed in larger, multimodal studies, but they support the potential role of iTBS-assisted rehabilitation for lower-limb dysfunction after stroke.

## Data Availability

The original contributions presented in the study are included in the article/Supplementary material, further inquiries can be directed to the corresponding author.
