# Supplementary material for: Functional near-infrared spectroscopy study of intermittent theta burst stimulation on lower-limb motor dysfunction in patients with post-stroke hemiplegia
Source: Front Hum Neurosci. 2026 Jun 22;20:1849251. doi: 10.3389/fnhum.2026.1849251 (PMC13333760; doi:10.3389/fnhum.2026.1849251)
Supplement: Supplementary file 1 [file Table_1.DOCX]

Supplementary table 1 Correlation coefficients between changes in HbO₂ (post treatment minus pre treatment) and changes in FMA LE scores in the treatment group.

| Channels | r value | p values |
| --- | --- | --- |
| S6-D6 | -0.081 | 0.653 |
| S6-D7 | 0.424 | 0.014 |
| S7-D7 | 0.361 | 0.039 |
| S7-D8 | -0.061 | 0.735 |
| S8-D8 | -0.202 | 0.260 |
| S9-D6 | -0.291 | 0.100 |
| S9-D7 | -0.375 | 0.031 |
| S10-D7 | 0.375 | 0.032 |
| S10-D8 | 0.040 | 0.823 |
| S11-D8 | 0.212 | 0.237 |
| S12-D9 | 0.159 | 0.376 |
| S13-D9 | -0.196 | 0.275 |
| S13-D10 | 0.044 | 0.808 |
| S14-D10 | -0.142 | 0.430 |
| S14-D11 | -0.116 | 0.521 |
| S15-D9 | 0.020 | 0.911 |
| S16-D9 | -0.483 | 0.004 |
| S16-D10 | 0.267 | 0.134 |
| S17-D10 | -0.039 | 0.831 |
| S17-D11 | 0.128 | 0.479 |

Supplementary table 2. Subgroup analysis of the female population: comparison between control and treatment groups after treatment.

|  | Treatment | Control | z | p |
| --- | --- | --- | --- | --- |
| Female | 15 | 16 |  |  |
| FMA-LE | 27(23.5-29.5) | 20(17.75-26.25) | -2.199 | 0.028 |
| S6D6 | 0.05(0.04-0.05) | 0.05(0.02-0.09) | 0.000 | 1.000 |
| S6D7 | 0.07(0.02-0.08) | 0.04(0.02-0.07) | -0.671 | 0.502 |
| S7D7 | 0.08(0.02-0.13) | 0.04(0.02-0.06) | -1.502 | 0.133 |
| S7D8 | 0.06(0.04-0.11) | 0.08(0.02-0.17) | -0.079 | 0.937 |
| S8D8 | 0.06(0.04-0.27) | 0.04(0.03-0.09) | -1.580 | 0.114 |
| S9D6 | 0.02(0.02-0.05) | 0.02(0.01-0.03) | -0.159 | 0.874 |
| S9D7 | 0.03(0.02-0.05) | 0.03(0-0.06) | -0.434 | 0.664 |
| S10D7 | 0.07(0.03-0.09) | 0.06(0.02-0.08) | -0.869 | 0.385 |
| S10D8 | 0.06(0.04-0.1) | 0.02(0.01-0.07) | -1.422 | 0.155 |
| S11D8 | 0.05(0.03-0.1) | 0.03(0.02-0.05) | -2.095 | 0.036 |
| S12D9 | 0.05(0.02-0.08) | 0.04(0.02-0.05) | -0.750 | 0.453 |
| S13D9 | 0.03(0.02-0.07) | 0.03(0.02-0.05) | -0.277 | 0.782 |
| S13D10 | 0.04(0.01-0.08) | 0.03(0.01-0.04) | -0.711 | 0.477 |
| S14D10 | 0.05(0.02-0.06) | 0.01(0.01-0.1) | -1.580 | 0.114 |
| S14D11 | 0.06(0.03-0.07) | 0.05(0.03-0.06) | -0.079 | 0.937 |
| S15D9 | 0.09(0.05-0.13) | 0.06(0.03-0.09) | -1.779 | 0.075 |
| S16D9 | 0.07(0.04-0.11) | 0.03(0.01-0.06) | -1.779 | 0.075 |
| S16D10 | 0.05(0.04-0.08) | 0.05(0.01-0.08) | -0.750 | 0.453 |
| S17D10 | 0.06(0.03-0.09) | 0.03(0.02-0.06) | -1.542 | 0.123 |
| S17D11 | 0.08(0.03-0.12) | 0.04(0.01-0.1) | -0.750 | 0.453 |

Supplementary table 3. Subgroup analysis of the male population: comparison between control and treatment groups after treatment.

|  | Treatment | Control | z | p |
| --- | --- | --- | --- | --- |
| Male | 18 | 17 |  |  |
| FMA-LE | 25(21.5-26) | 18(17-21) | -2.806 | 0.005 |
| S6D6 | 0.05(0.04-0.08) | 0.02(0.01-0.09) | -1.650 | 0.099 |
| S6D7 | 0.02(0.01-0.07) | 0.02(0.02-0.06) | -0.793 | 0.428 |
| S7D7 | 0.03(0.02-0.07) | 0.02(0.02-0.06) | 0.000 | 1.000 |
| S7D8 | 0.05(0.04-0.06) | 0.02(0.01-0.06) | -1.980 | 0.048 |
| S8D8 | 0.11(0.09-0.3) | 0.05(0.03-0.07) | -3.367 | 0.001 |
| S9D6 | 0.02(0.02-0.11) | 0.03(0.01-0.07) | -0.660 | 0.509 |
| S9D7 | 0.05(0.02-0.08) | 0.05(0.03-0.07) | -0.429 | 0.668 |
| S10D7 | 0.02(0.01-0.06) | 0.04(0-0.06) | -0.165 | 0.869 |
| S10D8 | 0.07(0.04-0.11) | 0.04(0.02-0.09) | -1.188 | 0.235 |
| S11D8 | 0.04(0.02-0.08) | 0.03(0.02-0.05) | -1.487 | 0.137 |
| S12D9 | 0.03(0.02-0.05) | 0.02(0.02-0.04) | -0.396 | 0.692 |
| S13D9 | 0.04(0.03-0.08) | 0.03(0.02-0.04) | -1.024 | 0.306 |
| S13D10 | 0.03(0.02-0.06) | 0.03(0.01-0.03) | -0.561 | 0.575 |
| S14D10 | 0.06(0.04-0.11) | 0.07(0.01-0.11) | -0.264 | 0.792 |
| S14D11 | 0.07(0.05-0.1) | 0.04(0.03-0.06) | -1.122 | 0.262 |
| S15D9 | 0.07(0.03-0.15) | 0.06(0.01-0.11) | -0.693 | 0.488 |
| S16D9 | 0.05(0.05-0.08) | 0.06(0.02-0.08) | -0.362 | 0.717 |
| S16D10 | 0.05(0.01-0.08) | 0.04(0.01-0.05) | -0.462 | 0.644 |
| S17D10 | 0.06(0.03-0.07) | 0.03(0.02-0.04) | -2.442 | 0.015 |
| S17D11 | 0.04(0.03-0.1) | 0.07(0.01-0.1) | -0.298 | 0.766 |

Supplementary table 4. Subgroup analysis of the cerebral infarction population: comparison between control and treatment groups after treatment.

|  | Treatment | Control | z | p |
| --- | --- | --- | --- | --- |
| Cerebral infarction | 18 | 19 |  |  |
| FMA-LE | 25.5(23.25-27.75) | 20(16.5-25) | -2.087 | 0.037 |
| S6D6 | 0.05(0.04-0.07) | 0.02(0.02-0.07) | -1.457 | 0.145 |
| S6D7 | 0.05(0.01-0.08) | 0.02(0.01-0.05) | -1.094 | 0.274 |
| S7D7 | 0.06(0.02-0.13) | 0.02(0.02-0.06) | -1.640 | 0.101 |
| S7D8 | 0.04(0.03-0.06) | 0.06(0.01-0.15) | -0.274 | 0.784 |
| S8D8 | 0.1(0.04-0.28) | 0.04(0.03-0.06) | -2.796 | 0.005 |
| S9D6 | 0.02(0.01-0.08) | 0.03(0.02-0.07) | -0.760 | 0.447 |
| S9D7 | 0.03(0.02-0.06) | 0.05(0.02-0.07) | -0.486 | 0.627 |
| S10D7 | 0.06(0.02-0.08) | 0.05(0.01-0.08) | -1.003 | 0.316 |
| S10D8 | 0.05(0.02-0.07) | 0.06(0.02-0.1) | -0.243 | 0.808 |
| S11D8 | 0.04(0.03-0.08) | 0.03(0.02-0.04) | -2.036 | 0.042 |
| S12D9 | 0.04(0.03-0.07) | 0.03(0.02-0.05) | -0.729 | 0.466 |
| S13D9 | 0.03(0.02-0.1) | 0.03(0.02-0.1) | -0.122 | 0.903 |
| S13D10 | 0.04(0.02-0.08) | 0.02(0.01-0.04) | -1.551 | 0.121 |
| S14D10 | 0.05(0.02-0.07) | 0.1(0.03-0.11) | -0.942 | 0.346 |
| S14D11 | 0.05(0.02-0.07) | 0.06(0.03-0.07) | -0.577 | 0.564 |
| S15D9 | 0.11(0.05-0.19) | 0.07(0.06-0.12) | -1.491 | 0.136 |
| S16D9 | 0.06(0.05-0.1) | 0.05(0-0.07) | -1.612 | 0.107 |
| S16D10 | 0.08(0.04-0.08) | 0.05(0.01-0.07) | -1.491 | 0.136 |
| S17D10 | 0.06(0.03-0.07) | 0.03(0.02-0.04) | -1.884 | 0.060 |
| S17D11 | 0.09(0.04-0.1) | 0.03(0.01-0.09) | -1.185 | 0.236 |

Supplementary table 5. Subgroup analysis of the cerebral hemorrhage population: comparison between control and treatment groups after treatment.

|  | Treatment | Control | z | p |
| --- | --- | --- | --- | --- |
| Cerebral hemorrhage | 15 | 14 |  |  |
| FMA-LE | 26(21.5-30) | 18.5(17.25-20.5) | -2.669 | 0.008 |
| S6D6 | 0.05(0.03-0.08) | 0.04(0.02-0.12) | -0.262 | 0.793 |
| S6D7 | 0.02(0.01-0.07) | 0.06(0.02-0.08) | -1.483 | 0.138 |
| S7D7 | 0.03(0.02-0.08) | 0.05(0.02-0.06) | -0.698 | 0.485 |
| S7D8 | 0.06(0.05-0.09) | 0.02(0.01-0.04) | -2.530 | 0.011 |
| S8D8 | 0.11(0.06-0.32) | 0.05(0.03-0.08) | -2.226 | 0.026 |
| S9D6 | 0.02(0.02-0.09) | 0.02(0.01-0.03) | -1.483 | 0.138 |
| S9D7 | 0.04(0.03-0.07) | 0.03(0.01-0.05) | -1.440 | 0.150 |
| S10D7 | 0.02(0.01-0.06) | 0.04(0.01-0.07) | -0.393 | 0.694 |
| S10D8 | 0.1(0.06-0.16) | 0.03(0.01-0.04) | -2.837 | 0.005 |
| S11D8 | 0.05(0.03-0.08) | 0.04(0.02-0.06) | -1.702 | 0.089 |
| S12D9 | 0.02(0.02-0.06) | 0.02(0.02-0.05) | -0.436 | 0.663 |
| S13D9 | 0.04(0.03-0.06) | 0.02(0.02-0.03) | -2.095 | 0.036 |
| S13D10 | 0.02(0.01-0.06) | 0.03(0.02-0.04) | -0.480 | 0.631 |
| S14D10 | 0.06(0.04-0.09) | 0.01(0-0.02) | -2.706 | 0.007 |
| S14D11 | 0.07(0.05-0.1) | 0.03(0.02-0.05) | -1.789 | 0.074 |
| S15D9 | 0.06(0.03-0.09) | 0.03(0.01-0.06) | -1.483 | 0.138 |
| S16D9 | 0.05(0.04-0.07) | 0.04(0.02-0.06) | -0.524 | 0.600 |
| S16D10 | 0.04(0.02-0.06) | 0.05(0.02-0.07) | -0.436 | 0.663 |
| S17D10 | 0.05(0.03-0.08) | 0.03(0.02-0.05) | -1.789 | 0.074 |
| S17D11 | 0.04(0.02-0.09) | 0.09(0.01-0.1) | -0.305 | 0.760 |
